# Cardiovascular Response Patterns to Sympathetic Stimulation by Central Hypovolemia

**DOI:** 10.3389/fphys.2016.00235

**Published:** 2016-06-20

**Authors:** Anne-Sophie G. T. Bronzwaer, Jasper Verbree, Wim J. Stok, Mark A. van Buchem, Mat J. A. P. Daemen, Matthias J. P. van Osch, Johannes. J. van Lieshout

**Affiliations:** ^1^Department of Internal Medicine, Academic Medical Center, University of AmsterdamAmsterdam, Netherlands; ^2^Laboratory for Clinical Cardiovascular Physiology, Center for Heart Failure Research, Academic Medical CenterAmsterdam, Netherlands; ^3^Department of Radiology, Leiden University Medical CenterLeiden, Netherlands; ^4^Department of Anatomy, Embryology and Physiology, Academic Medical Center, University of AmsterdamAmsterdam, Netherlands; ^5^Department of Pathology, Academic Medical Center, University of AmsterdamAmsterdam, Netherlands; ^6^MRC/Arthritis Research UK Centre for Musculoskeletal Ageing Research, School of Life Sciences, University of Nottingham Medical School, Queen's Medical CentreNottingham, UK

**Keywords:** autonomic blood pressure control, central hypovolemia, baroreflex sensitivity, cardiovascular response patterns, lower body negative pressure

## Abstract

In healthy subjects, variation in cardiovascular responses to sympathetic stimulation evoked by submaximal lower body negative pressure (LBNP) is considerable. This study addressed the question whether inter-subject variation in cardiovascular responses coincides with consistent and reproducible responses in an individual subject. In 10 healthy subjects (5 female, median age 22 years), continuous hemodynamic parameters (finger plethysmography; Nexfin, Edwards Lifesciences), and time-domain baroreflex sensitivity (BRS) were quantified during three consecutive 5-min runs of LBNP at −50 mmHg. The protocol was repeated after 1 week to establish intra-subject reproducibility. In response to LBNP, 5 subjects (3 females) showed a prominent increase in heart rate (HR; 54 ± 14%, *p* = 0.001) with no change in total peripheral resistance (TPR; *p* = 0.25) whereas the other 5 subjects (2 females) demonstrated a significant rise in TPR (7 ± 3%, *p* = 0.017) with a moderate increase in HR (21 ± 9%, *p* = 0.004). These different reflex responses coincided with differences in resting BRS (22 ± 8 vs. 11 ± 3 ms/mmHg, *p* = 0.049) and resting HR (57 ± 8 vs. 71 ± 12 bpm, *p* = 0.047) and were highly reproducible over time. In conclusion, we found distinct cardiovascular response patterns to sympathetic stimulation by LBNP in young healthy individuals. These patterns of preferential autonomic blood pressure control appeared related to resting cardiac BRS and HR and were consistent over time.

## Introduction

Lower body negative pressure (LBNP) is used in research settings as a model to study the cardiovascular effects of central hypovolemia in humans (Hinojosa-Laborde et al., 2014). Application of sub-atmospheric pressure to the lower body redistributes fluid from the upper parts of the body into the compliant compartment of the lower extremities, leading to a decrease in venous return, and central blood volume (Cooke et al., 2004). Central blood volume is important for filling of the heart and directly affects stroke volume (SV) and cardiac output (CO). In response to a progressive reduction of central blood volume as elicited by LBNP, both SV and CO decrease modifying arterial pulse pressure and its pulsatility (Michard et al., 2000; Bronzwaer et al., 2015). This results in a baroreceptor mediated reflex increase in heart rate (HR) and total peripheral (vascular) resistance (TPR) (Schadt and Ludbrook, 1991; Ryan et al., 2012). A reduction in central blood volume evoked by LBNP or posture changes (e.g., standing up) elicits a wide range of HR and blood pressure (variation) responses among healthy individuals (Smith, 1990; Ramirez-Marrero et al., 2008; Ryan et al., 2010; Bronzwaer et al., 2014). Studies addressing the cardiovascular responses and specifically tolerance to a reduction in central blood volume evoked by LBNP reported that tolerance time and cardiovascular responses were reproducible in a test-retest condition at varying time intervals (Lightfoot et al., 1991; Convertino, 2001; Howden et al., 2001; Lee et al., 2004; Kay and Rickards, 2015). From observations in our lab we found considerable differences in cardiovascular response patterns to LBNP between subjects. Only a few studies have specifically addressed individual response patterns from rest to maximal LBNP into some detail (Batzel et al., 2009; Goswami et al., 2009). We questioned whether the large variation in response patterns between subjects to submaximal LBNP coincides with consistent and reproducible responses in an individual subject.

Therefore, the present study was designed to evaluate the individual cardiovascular reflex responses to sympathetic stimulation and their robustness. To that purpose, we determined intra-subject reproducibility of responses by central hypovolemia evoked by LBNP in young healthy subjects over short (5 min) and longer (1 week) time intervals.

## Methods

### Subjects

Ten healthy, non-smoking Caucasian subjects (5 females), with normal physical fitness and with a median (range) age of 22 (19–26) year, height of 174 (166–177) cm, and weight 69 (55–77) kg participated in this study. Exclusion criteria included a medical history of cardio- and/or cerebrovascular disease, neurological disorders, diabetes mellitus, regular fainting, and the use of medication (either prescription or non-prescription). Subjects abstained from heavy exercise and caffeinated beverages 5 h prior to the experiment. Phase of menstrual cycle in female subjects was not accounted for. Experiments were conducted in a temperature-controlled laboratory (20–22°C) at the same time of the day (12–4 p.m.) to avoid potential effects of circadian rhythm on the study outcomes. The institutional Medical Ethics Committee approved the protocol and written informed consent was obtained.

### Experimental protocol

Measurements were performed in a quiet room with subjects in the supine position. After instrumentation, the lower body was positioned inside the LBNP box (Dr. Kaiser Medizintechnik, Bad Hersfeld, Germany) and sealed at the level of the iliac crest (Goswami et al., 2009). The study protocol included 5 min of rest, followed by three 5 min trials of LBNP at −50 mmHg separated by 5 min of rest. During the experiment, subjects were instructed to breathe normally and to avoid body movement. Reproducibility of cardiovascular responses was evaluated by repeating the protocol 7 days later.

The LBNP-box was equipped with a saddle to avoid leg muscle pump activation during the application of sub-atmospheric pressure. The pressure inside the box was manually controlled and established within 10–20 s. LBNP was terminated upon request or in case of (pre-)syncopal symptoms which were determined by one or more of the following criteria: systolic arterial pressure (SAP) below 80 mmHg, or rapid drop (SAP by ≥20 mmHg/min, diastolic (DAP) by ≥10 mmHg/min), drop in HR by ≥15 bpm, and/or sweating, light-headedness, nausea, blurred vision, or skin pallor.

### Measurements and analysis

Continuous arterial pressure (AP) was measured non-invasively by a volume clamp method using finger plethysmography (Nexfin, Edwards Lifesciences BMEYE, the Netherlands). HR was expressed as the inverse of the inter-beat interval. Left ventricular stroke volume (SV) and cardiac output (CO; SV multiplied by instantaneous HR) were measured by a pulse contour method (Nexfin CO-trek, Edwards Lifesciences BMEYE, Amsterdam, the Netherlands) which is validated against thermodilution estimates of CO (Bogert et al., 2010; Truijen et al., 2012b). TPR was defined as the ratio of mean arterial pressure (MAP) and CO. All recorded signals were visually inspected for artifacts and analyzed offline (Matlab R2007b, Mathworks Inc. MA, USA).

Time-domain cardiac baroreflex sensitivity (BRS) was analyzed using the cross-correlation method (Westerhof et al., 2004; Gisolf et al., 2005). First, beat-to-beat SAP and inter-beat interval (IBI) were fitted with cubic spline functions and resampled at 1 s intervals. The cross-correlation between 10 s series of resampled SAP and IBI signals were computed for various delays (τ) in IBI of 0–5 s. The delay between SAP and IBI with the highest cross-correlation was selected if the correlation was significant at *p* < 0.05. The regression slope was recorded as one BRS value together with the τ. Subsequently, the process was repeated by shifting the 10 s window. An average value of BRS was calculated over the last minute of rest and LBNP.

### Statistical analysis

Variables were presented as mean ± SD. One-way repeated measures ANOVAs were used to compare the last minute of rest and the last minute of LBNP across three consecutive trials on day 0 and 7, followed by Holm-Sidak's *post hoc* tests. The responses were grouped together per day when there were no differences in baseline and LBNP responses across trials. The effect of LBNP on the measured parameters was analyzed with a paired two-tailed Student's *T*-test (Sigmaplot 11.0, Systat Software Inc., USA) comparing the last minute of rest with the last minute of LBNP (average of three trials). Intraclass correlation coefficients (ICC) and coefficients of variation (CV) were calculated (IBM SPSS statistics 20, IBM corporation, USA) to assess intra-subject reproducibility. Intra-subject reproducibility was evaluated across LBNP trials (5 min periods; three trials on day 0) and sessions (1 week period; average response of three trials between day 0 and day 7). One trial was defined as the last 3 min of rest, 5 min of LBNP and 2 min of recovery. ICC and CV values were calculated per trial time point and then averaged for all time points. No universal standard exists for classifying ICC and tests of statistical significance of reproducibility measures are of little practical utility (Morrow and Jackson, 1993). Therefore, reproducibility was defined as poor if ICC < 0.40, acceptable if coefficients ranged from 0.41 to 0.60, good if coefficients ranged from 0.61 to 0.80 and excellent if ICC ≥ 0.81 (Landis and Koch, 1977). Others have adopted these criteria in assessing reproducibility of HR variability at rest (Marks and Lightfoot, 1999) and in response to LBNP (Lee et al., 2004). Intra-subject reproducibility was defined good if group average CV < 10%. A *p* < 0.05 was considered to indicate a statistically significant difference.

## Results

A total of 60 LBNP trials were performed (three trials per subject per measurement day). Six LBNP trials were prematurely aborted due to a sudden drop in blood pressure (*N* = 2, different subjects), insufficient quality of Nexfin signals (*N* = 2, same subject) or failure to reach the required LBNP pressure level (*N* = 2, same subject). As a result, 54 LBNP trials entered final analysis. In a separate trial, integrity of the cardiovascular autonomic function was verified by passive head-up tilt testing (data not shown).

### Group response

**Figure 1:**
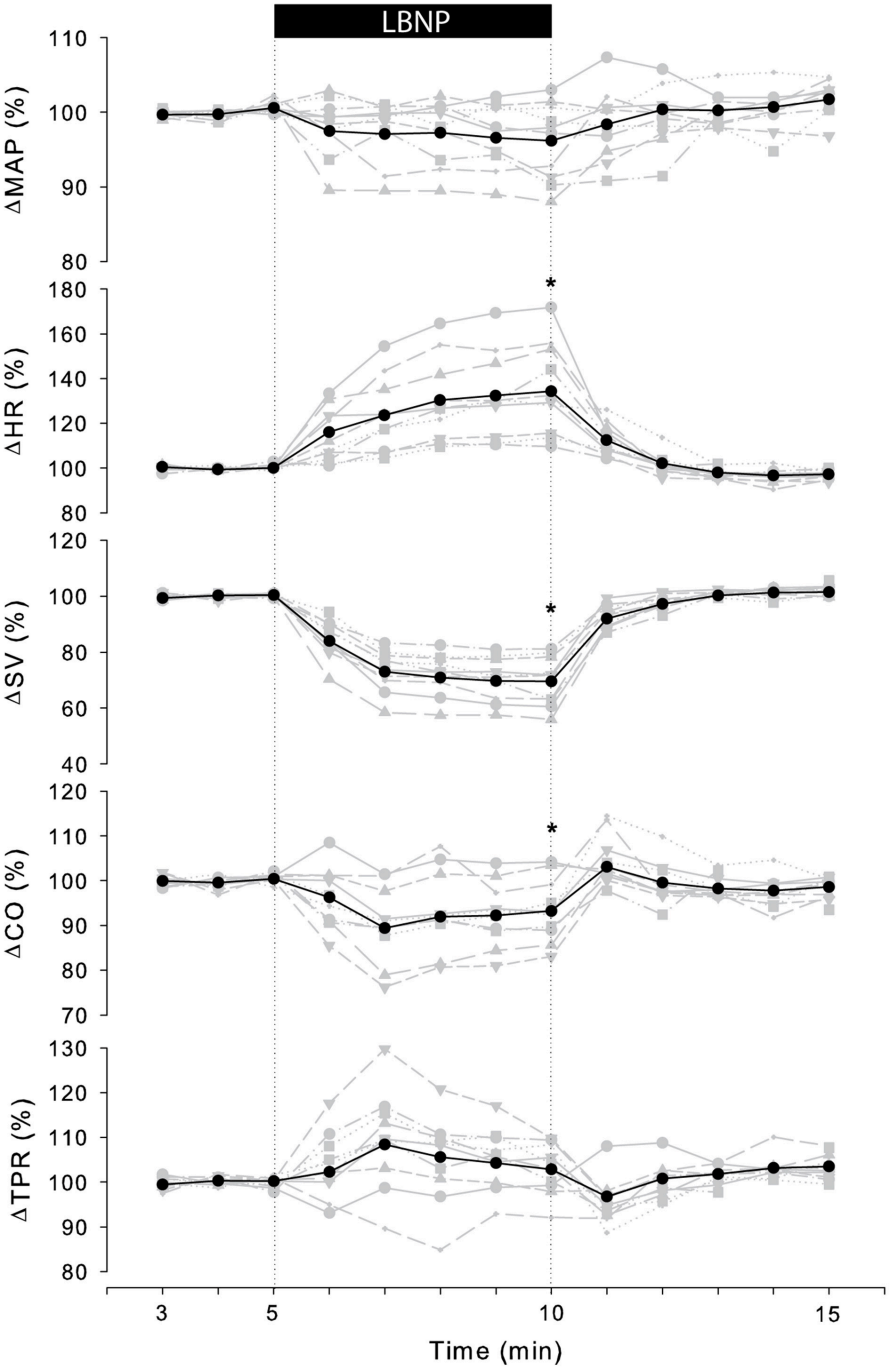
**Individual (gray) and averaged (black) hemodynamic responses to LBNP**. Data was normalized to the last 2 min of rest. MAP, mean arterial pressure; HR, heart rate; SV, stroke volume; CO, cardiac output; TPR, total peripheral resistance. ^*^p < 0.05 last min of LBNP vs. last min of rest.

Table 1 summarizes the averaged cardiovascular response to 5 min of −50 mmHg LBNP for three consecutive trials measured on day 0 and 7. There were no significant differences in absolute values between consecutive trials at rest or in response to LBNP such that responses were grouped together per day in further analysis. Figure 1 (black line) shows the normalized group response to LBNP for day 0. HR (37 ± 21%, *p* < 0.001) increased with a fall in systolic (SAP; −11 ± 6%, *p* < 0.001) and mean arterial pressure (MAP; −5 ± 5%, *p* = 0.02), SV (−31 ± 9%, *p* < 0.001) and CO (−6 ± 7%, *p* = 0.02). Diastolic (DAP; *p* = 0.25) pressure as well as TPR (*p* = 0.17) did not change. BRS decreased (−50 ± 13%, *p* < 0.001) in response to LBNP.

**Table 1:** **Hemodynamic response to LBNP for three consecutive trials at day 0 and day 7**.

			**Trial 1**		**Trial 2**		**Trial 3**		**Trial 1 vs. 2 vs. 3 (*p*-values)**	
			***Rest***	***LBNP***	***Rest***	***LBNP***	***Rest***	***LBNP***	***Rest***	***LBNP***
SAP	(mmHg)	day 0	120 ± 15	109 ± 14^*^	121 ± 14	107 ± 13^*^	123 ± 12	109 ± 11^*^	0.670	0.237
		day 7	116 ± 10	102 ± 5^*^	115 ± 9	104 ± 4^*^	118 ± 8	104 ± 4^*^	0.194	0.245
DAP	(mmHg)	day 0	69 ± 12	71 ± 10	69 ± 10	69 ± 9	70 ± 9	71 ± 7	0.772	0.096
		day 7	67 ± 6	67 ± 2	67 ± 5	68 ± 4	68 ± 5	68 ± 3	0.191	0.145
MAP	(mmHg)	day 0	88 ± 14	85 ± 12^*^	89 ± 12	83 ± 10^*^	89 ± 10	85 ± 8^*^	0.773	0.124
		day 7	85 ± 7	80 ± 3^*^	84 ± 6	81 ± 4^*^	86 ± 6	81 ± 3^*^	0.124	0.124
HR	(beats/min)	day 0	66 ± 13	90 ± 10^*^	64 ± 12	85 ± 12^*^	62 ± 13	87 ± 10^*^	0.067	0.432
		day 7	62 ± 13	83 ± 14^*^	60 ± 12	83 ± 15^*^	60 ± 12	82 ± 16^*^	0.137	0.496
SV	(ml)	day 0	112 ± 16	78 ± 16^*^	113 ± 17	78 ± 17^*^	114 ± 17	79 ± 16^*^	0.233	0.810
		day 7	111 ± 15	76 ± 13^*^	109 ± 12	73 ± 12^*^	110 ± 12	75 ± 11^*^	0.476	0.326
CO	(l/min)	day 0	7.1 ± 1.3	6.8 ± 1.2^*^	7 ± 1	6.5 ± 1.1^*^	6.9 ± 1.1	6.6 ± 1.1^*^	0.435	0.098
		day 7	6.4 ± 1.1	6.3 ± 0.9	6.5 ± 1.1	6.1 ± 0.7^*^	6.5 ± 1.2	6.1 ± 0.9^*^	0.976	0.178
TPR	(dyn.sec/cm^5^)	day 0	1000 ± 152	1048 ± 204	1023 ± 180	1033 ± 172	1039 ± 164	1026 ± 172	0.081	0.156
		day 7	1017 ± 176	1042 ± 140	1046 ± 184	1086 ± 142	1045 ± 180	1078 ± 156	0.497	0.510
BRS	(ms/mmHg)	day 0	17 ± 8	8 ± 4^*^	19 ± 8	8 ± 2^*^	17 ± 7	8 ± 3^*^	0.089	0.765
		day 7	17 ± 6	10 ± 3^*^	20 ± 7	10 ± 3^*^	20 ± 10	10 ± 4^*^	0.419	0.877

*Values are presented as mean ± SD. AP, arterial pressure (Systolic, Diastolic, and Mean); HR, heart rate; SV, stroke volume; CO, cardiac output; TPR, total peripheral resistance; BRS, baroreflex sensitivity. Rest and LBNP response were compared between Trial 1, Trial 2, and Trial 3*.

* *p < 0.05 vs. rest*.

### Individual responses

**Figure 2:**
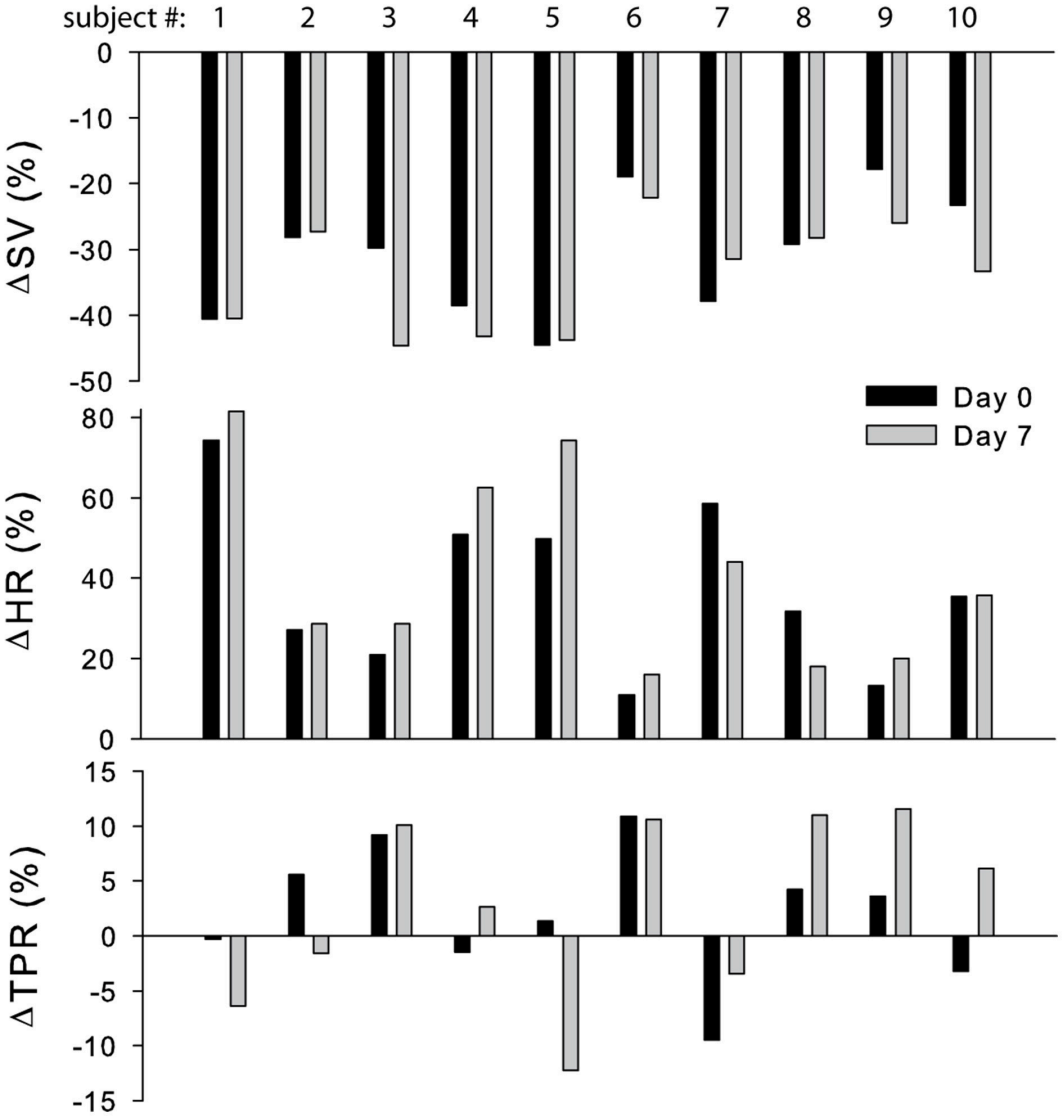
**Distribution of maximal change in SV, HR, and TPR in response to LBNP at day 0 (black bars) and day 7 (gray bars)**. SV, stroke volume; HR, heart rate; TPR, total peripheral resistance.

**Figure 3:**
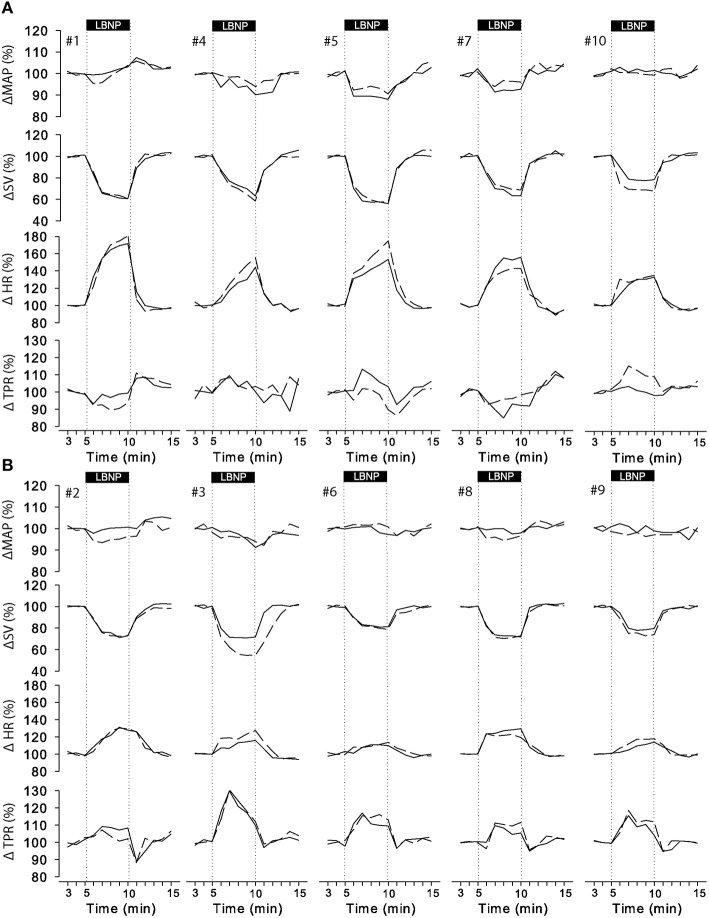
**Individual responses to LBNP for day 0 (solid line) and day 7 (dashed line)**. Data was normalized to the last 2 min of rest. Group A (**A**, upper panel) showed a predominant increase in HR whereas group B (**B**, lower panel) responded by a consistent increase in TPR with smaller change in HR. MAP, mean arterial pressure; SV, stroke volume; HR, heart rate; TPR, total peripheral resistance.

**Figure 4:**
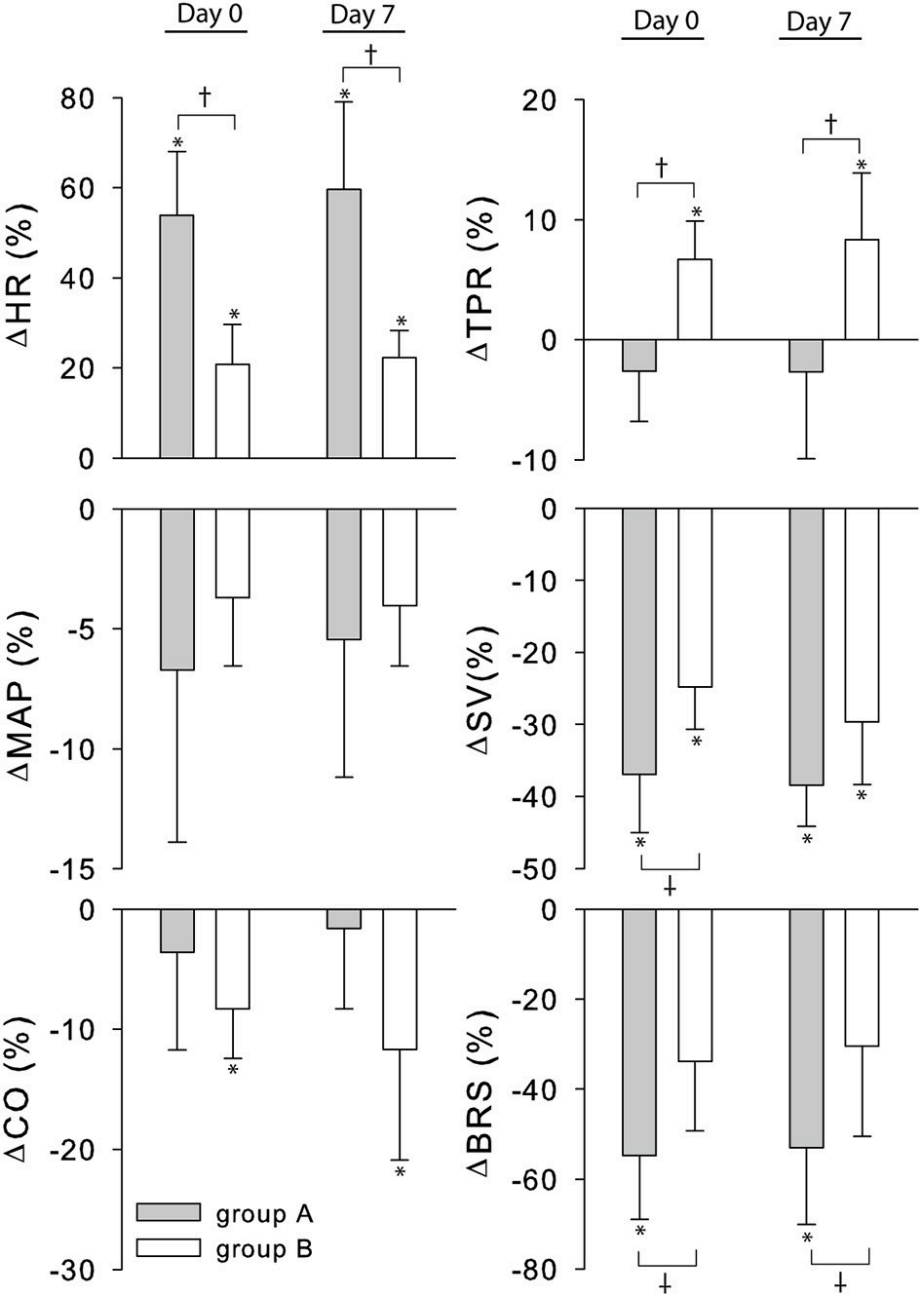
**Percent change from rest to LBNP for group A (gray bars) and B (white bars)**. MAP, mean arterial pressure; CO, cardiac output; SV, stroke volume; HR, heart rate; TPR, total peripheral resistance; BRS, baroreflex sensitivity. ^*^p < 0.05 rest vs. LBNP ^†^*p* < 0.05 group A vs. group B.

**Figure 5:**
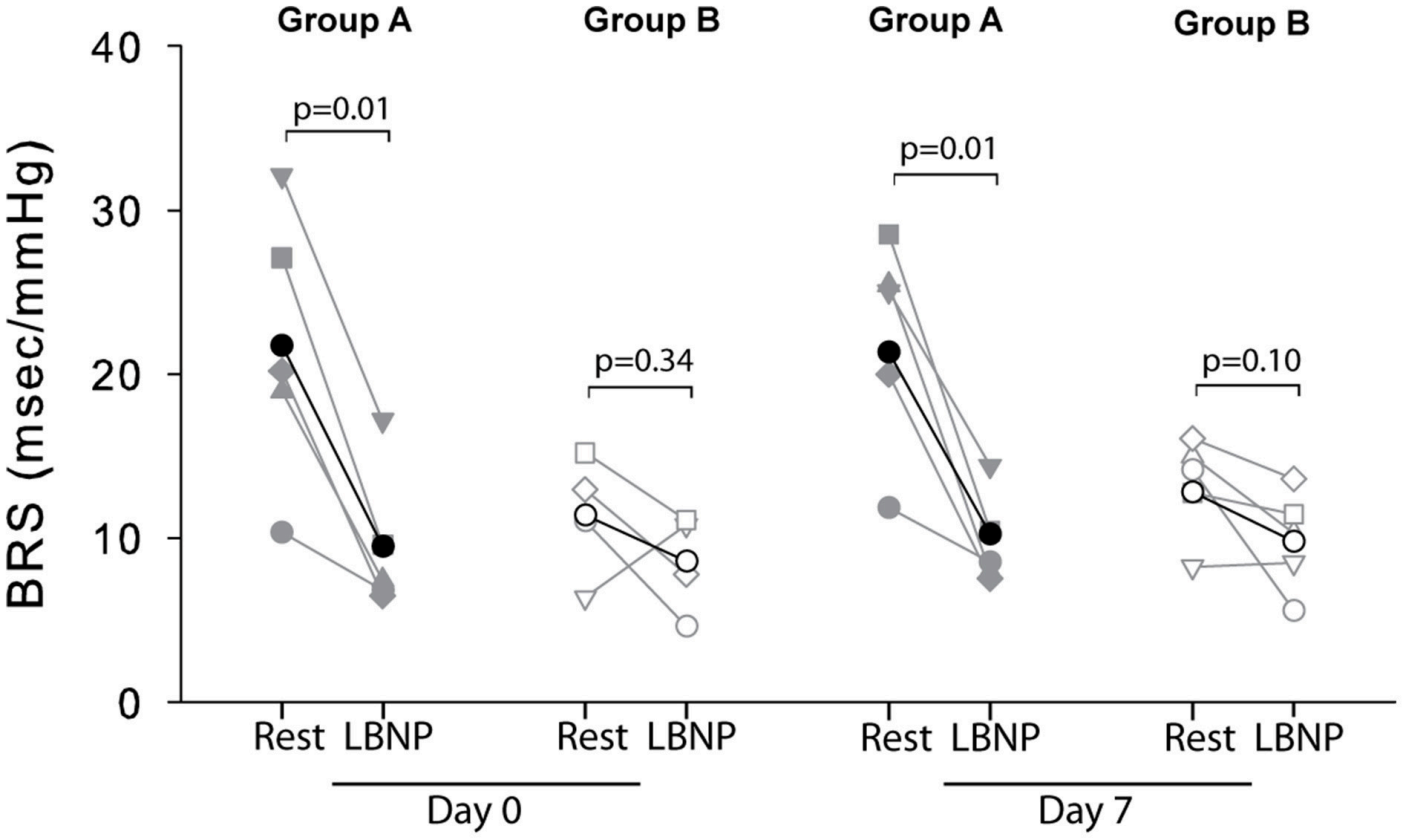
**Individual (gray) and average (black) BRS responses to LBNP for group A (filled symbols) and group B (open symbols) at day 0 and day 7**. No BRS values could be determined for subject 2 (group B) at day 0 due to signal artifacts.

**Figure 6:**
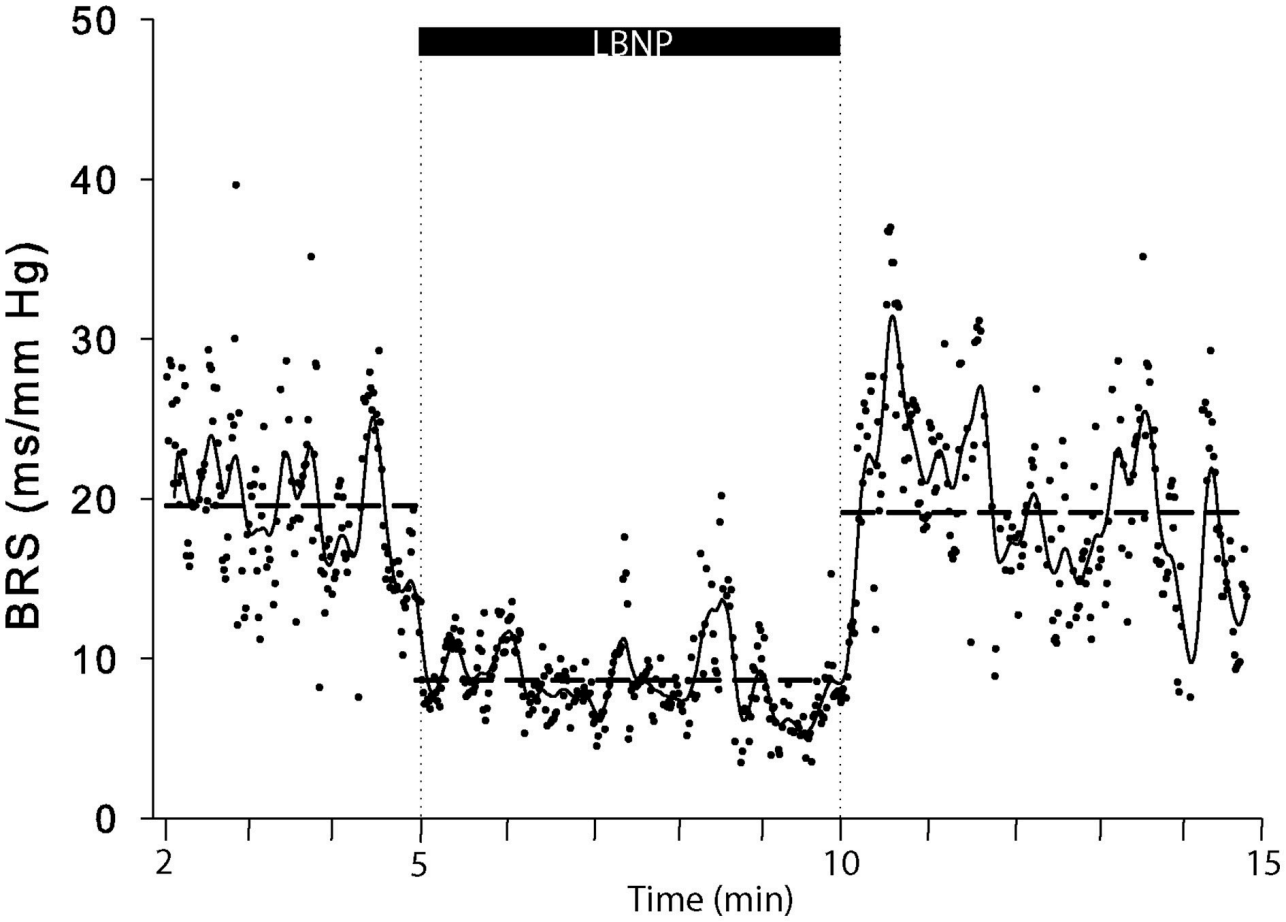
**A representative example (subject 5) of BRS computations in response to LBNP**. Each dot represents a BRS result, drawn horizontal dashed lines represent period averages and the solid line represents moving averages.

The cardiovascular compensatory response to LBNP differed between subjects (Figure 1, gray lines). Figure 2 gives the distribution of maximal changes in HR, SV, and TPR. Subsequently, subjects were dichotomized into two equal-sized groups (A and B) based on the median change in HR (Figure 3 and Table 2). Group A showed a prominent increase in HR (54 ± 14%, *p* = 0.001) with no significant change in TPR (*p* = 0.25) vs. group B demonstrating a moderate increase in HR (21 ± 9%, *p* = 0.004) and a rise in TPR (7 ± 3%, *p* = 0.017). The response pattern of group A vs. B coincided with a larger decrease in SV (−37 ± 8 vs. −25 ± 6%, *p* = 0.026) and BRS (−54 ± 14 vs. −34 ± 15%, *p* = 0.036) see Figures 4, 5. Group A vs. B subjects did not differ in sex, age, or body mass index (BMI), however lower resting HR (57 ± 8 vs. 71 ± 12 bpm, *p* = 0.047) and higher resting BRS (22 ± 8 vs. 11 ± 3 ms/mmHg, *p* = 0.049) were found in group A. Figure 6 shows BRS results of one representative subject.

**Table 2:** **Baseline characteristics and hemodynamic response to LBNP for group A and group B**.

			**Group A (*n* = 5)**		**Group B (*n* = 5)**	
			***Rest***	***LBNP***	***Rest***	***LBNP***
Sex	(M/F)		2/3		3/2	
Age	(years)		21 ± 2		24 ± 2	
BMI	(kg/m^2^)		23 ± 3		23 ± 3	
SAP	(mmHg)	day 0	123 ± 10	106 ± 14^*^	120 ± 17	110 ± 13^*^
		day 7	117 ± 8	102 ± 4^*^	116 ± 9	104 ± 4^*^
DAP	(mmHg)	day 0	67 ± 7	68 ± 8	71 ± 14	71 ± 12
		day 7	68 ± 4	69 ± 2	67 ± 6	67 ± 6
MAP	(mmHg)	day 0	87 ± 7	82 ± 10	88 ± 16	87 ± 14
		day 7	86 ± 5	81 ± 2	84 ± 7	81 ± 5
HR	(beats/min)	day 0	57 ± 8	87 ± 13^*^	71 ± 12^†^	86 ± 12^*^
		day 7	55 ± 7	88 ± 14^*^	67 ± 13^†^	81 ± 17^*^
SV	(ml)	day 0	121 ± 13	77 ± 17^*^	105 ± 17	79 ± 16^*^
		day 7	113 ± 16	70 ± 13^*^	108 ± 10	76 ± 13^*^
CO	(l/min)	day 0	6.8 ± 1.1	6.6 ± 1.3	7.3 ± 1.2	6.6 ± 1^*^
		day 7	6.1 ± 1.1	6.0 ± 0.8	6.8 ± 1.1	6.0 ± 0.8^*^
TPR	(dyn.sec/cm^5^)	day 0	1042 ± 80	1015 ± 89	994 ± 213	1062 ± 261^*^
		day 7	1149 ± 160	1111 ± 105	977 ± 187	1100 ± 179^*^
BRS	(ms/mmHg)	day 0	22 ± 8	10 ± 4^*^	11 ± 4^†^	9 ± 4
		day 7	22 ± 7	10 ± 3^*^	13 ± 3^†^	10 ± 3

*Values are presented as mean ± SD. BMI, body mass index; AP, arterial pressure (Systolic, Diastolic and Mean,); HR, heart rate; SV, stroke volume; CO, cardiac output; TPR, total peripheral resistance; BRS, baroreflex sensitivity*.

* *p < 0.05 vs. rest*.

† *p < 0.05 vs. group A*.

### Reproducibility

Figure 3 (dashed lines) visually demonstrated that the majority of individuals responded similarly 1 week later. Intra-subject reproducibility of the cardiovascular response to LBNP according to the ICC and the CV is given in Table 3. HR, SV, CO, and TRP demonstrated good to excellent intra-subject reproducibility (ICC ≥ 0.61 and CV < 10%) for both short-term (5 min) and long-term (1 week) repeats. The intra-subject reproducibility of arterial pressure (SAP, DAP and MAP) responses across trials and sessions was poor according to the ICC (i.e., ≤ 0.40) but good according to CV with DAP just falling outside the criteria of 10% CV.

**Table 3:** **Intra-subject reproducibility in response to LBNP**.

		**ICC**		**CV**	
		**Across trials**	**Across sessions**	**Across trials**	**Across sessions**
SAP	(mmHg)	0.38 ± 0.43	0.28 ± 0.24	8.2 ± 5.3	7.8 ± 7.4
DAP	(mmHg)	0.29 ± 0.41	0.35 ± 0.32	13.4 ± 15.9	12.4 ± 18.9
MAP	(mmHg)	0.37 ± 0.35	0.56 ± 0.37	10.2 ± 10.3	9.1 ± 9.6
HR	(beats/min)	0.87 ± 0.10	0.92 ± 0.05	4.2 ± 3.2	4.5 ± 3.7
SV	(mL)	0.97 ± 0.02	0.96 ± 0.04	2.8 ± 4.1	4.1 ± 5.2
CO	(L/min)	0.72 ± 0.20	0.70 ± 0.21	4.5 ± 6.8	6.1 ± 7.5
TPR	(dyn.sec/cm^5^)	0.67 ± 0.21	0.67 ± 0.29	4.3 ± 7.5	7.9 ± 8.2

*Intra-class correlation coefficients (ICC) and coefficients of variation (%CV) representing intra-subject reproducibility across three trials and two sessions. Data presented as mean ± SD. AP, arterial pressure (Systolic, Diastolic and Mean); HR, heart rate; SV, stroke volume; CO, cardiac output; TPR, total peripheral resistance*.

## Discussion

The findings of the present study provide important information regarding autonomic blood pressure control in humans. We observed distinct cardiovascular response patterns to sympathetic stimulation by LBNP in young healthy individuals. These patterns of preferential autonomic blood pressure control appeared related to resting cardiac BRS and HR. The finding that these patterns within an individual were consistent over a week suggest a programmed reflex response to sympathetic stimulation.

The major neural pathway for acute BP regulation involves baroreflex function (Rowell, 1986; Monahan, 2007). In response to LBNP, blood is redistributed from the chest into the lower parts of the body, which is largely contained in the venous compartment and so does not contribute effectively to the circulating blood volume (Sjöstrand, 1953; Rowell, 1986). Traditionally, the first autonomic cardiovascular response to such a reduction in cardiac preload is believed to be represented by a fast and predominant increase in HR corresponding to vagal withdrawal as the first line of defense. Arterial blood pressure is maintained further by enhancement of sympathetic influence on both HR and TPR which occurs more slowly due to a longer time-constant (Lanfranchi and Somers, 2002; Shaffer et al., 2014). Recently, a more balanced model of sympatho-vagal control representing a continuous interplay between vagal and sympathetic modulation of HR has been proposed without clear on/off thresholds (White and Raven, 2014). In humans, the relative contribution of arterial vs. cardiopulmonary baroreflex involvement cannot be ascertained; for instance, even mild LBNP reduces aortic dimensions contesting selective low pressure area receptor activation (Taylor et al., 1995). The present study demonstrated two qualitatively different cardiovascular reflex patterns in response to a similar degree of exposure of LBNP varying from a predominant effect on HR to a consistent increase in TPR with a smaller change in HR. These differential responses between HR and TPR coincided with a larger decline in SV and CO. This conforms to data from Fu et al. (2004a) who raised the hypothesis that decreases in pulse amplitude (a function of SV) may preferentially influence the vagal component of the baroreflex, whereas flow in baroreceptive arteries (a function of CO) dominates the sympathetic component. In addition, our data show that different reflex responses coincided with resting values of dynamic baroreflex control and HR. Together with an insubstantial increase in TPR in subjects with higher resting BRS, this alludes to differential cardiovascular reflex control in response to simulated central hypovolemia. The observed variance in responses was reproducible for the individual subjects suggesting an individually determined autonomic reflex response. We consider that humans may present with an identical sigmoidal baroreflex relationship as estimated by neck cuff suction-pressure plots but nevertheless may deliver different BRS values depending on the operating set point (Raven et al., 2005). Yielding multiple BRS values per minute rather than a single value reduces the risk of inaccurate reflections of baroreflex sensitivity which we consider a strength of the cross-correlation method used in the present study (see Figure 6).

Generally, subjects with high tolerance to central hypovolemia display signs and symptoms of greater sympathetic activition, e.g., higher HR and peripheral vasoconstriction with elevated neurohormonal activation (Convertino and Sather, 2000; Rickards et al., 2011; Convertino et al., 2012; Carter et al., 2016). Specifically, Convertino et al. demonstrated greater increases in muscle sympathetic nerve activity (MSNA) with an elevated total peripheral resistance and also an elevated HR, higher baseline cardiovagal BRS, and greater reductions in cardiovagal BRS in individuals with high tolerance to LBNP (Convertino et al., 2012). The magnitude of the autonomic responses to LBNP has been defined as the HR and vasoconstrictor “reserve” according to the concept that a greater physiological reserve capacity for tachycardia and vasoconstriction related to high tolerance to central hypovolemia is associated with greater reserves for sympathoexcitation and cardiac vagal withdrawal (Schondorf and Wieling, 2000; Fu et al., 2004b; Convertino et al., 2012). We consider that the present study addressed the cardiovascular responses to sub-maximal LBNP, i.e., beyond the “compensatory reserve” (Convertino et al., 2016). It appears as though subjects in group B may have a lower “HR reserve” and “BRS reserve” due to a higher resting HR and lower resting BRS. We did not determine the reproducibility of responses to maximal LBNP leaving the question how these responses might affect tolerance to maximal LBNP.

Of interest, in a retrospective study in a seemingly homogenous population with high tolerance to central hypovolemia a higher HR, TPR, SNA, and BRS appeared not associated with greater tolerance to a reduced central blood volume (Carter et al., 2016). This suggests that the autonomic make-up determines whether an individual relies on cardiac filling and vagal withdrawal to defend arterial pressure, or on sympathoexcitation to elevate HR and TPR (Carter et al., 2016). These findings were interpreted as to demonstrate the existence of subpopulations with analogous physiological abilities though diverse contributions of cardiovascular compensatory mechanisms to central blood volume depletion, and the present data conform to that concept. The recent observation that those subjects with high tolerance to central hypovolemia appear to be protected by maintained frontal lobe cortical tissue oxygen saturation links cerebral oxygen supply directly to brain function (Kay and Rickards, 2016).

Cardiovascular control is subjected to considerable environmental influences including level of deconditioning, hydration status, and disease (Butler et al., 1991; Levine et al., 1991; Schroeder et al., 2002; Truijen et al., 2010). Resting HR is a determinant of BRS (Kardos et al., 2001) and physiological factors, particularly age and sex, have significant impact on BRS in healthy subjects (Laitinen et al., 1998; Kardos et al., 2001).

### Age and sex

With aging the magnitude of the reflex increase in HR declines with BP maintained by a more substantial increase in forearm and total peripheral resistance (Ebert et al., 1982). Regarding a gender effect on the response to sympathetic stimulation, the available data on vascular responsiveness as well as on changes in HR and TPR during passive head-up tilt and/or LBNP is not uniform (Frey and Hoffler, 1988; Shoemaker et al., 2001). A gender effect on the vascular but not the HR response to LBNP has been reported with lesser increase in TPR in women (Frey and Hoffler, 1988). This conforms to recent data showing that a strong association between MSNA and TPR is expressed in young males only (Hart et al., 2012). In contrast, Shoemaker et al. demonstrated gender-related differences in HR and MSNA burst frequency but not in TPR in response to head-up tilt (Shoemaker et al., 2001). An influence of age does not apply to the participants in this study. We however cannot exclude that the sample size may have obscured a possible sex effect but we do consider that sex differences in baroreflex BP control during carotid hypotension have not been established (Kim et al., 2011).

### Physical fitness

An influence of differences in physical fitness level could be considered. We did not quantify maximal oxygen uptake but included subjects with normal physical fitness but without specific sports training as documented by a questionnaire. Exercise training does not affect vagal-cardiac control or cardiovagal BRS in young and middle-aged healthy subjects (Loimaala et al., 2000; Cooke and Carter, 2005). Accordingly, the reduction in resting HR by exercise training in young and middle-aged adults is limited (~ −5 bpm) (Levy et al., 1998; Loimaala et al., 2000) and does not account for the more than two-fold difference in resting HR between the two groups. The consistency of distinct cardiovascular response patterns as well as BRS with both autonomic response patterns maintaining blood pressure within the timeframe of the simulated central hypovolemia rather suggests an individually programmed strategy of reflex responses to sympathetic stimulation.

### Genetic vs. environmental

MSNA is considered as the primary index of sympathetic activity in humans (Fagius and Wallin, 1993; Joyner et al., 2010) and is characterized by large inter-individual differences but robust intra-individual reproducibility over many years which is in support of a genetic component (Fagius and Wallin, 1993). Kardos et al. showed that only half of the variance in BRS is attributable to simple anthropometric variables and common risk factors like smoking and alcohol consumption (Kardos et al., 2001). They suggested that the remaining variability reflects the subjects' different genetic background. In contrast, the significant degree of variance in cardiovascular responses to head-up tilt in identical twins suggests environmental respectively epigenetic factors as important contributing factors (O'Leary et al., 2006) that were not addressed in the present study.

### Methodological considerations

Several methodological considerations pertain to our data inherent to the study design. We do not directly measure individual differences in actual magnitude of the evoked fluid shift with LBNP. Inter-individual differences in SV are strongly correlated to central venous pressure (Johnson et al., 2014), so may reflect these fluid shifts, with subsequent consequences for baroreceptor reflex responses (Chapleau and Abboud, 1989). The inter-individual differences in central hypovolemia expressed as the LBNP-induced reduction in SV, seem a confounding factor related to the differential cardiovascular reflex responses between groups. The degree of central hypovolemia during LBNP is considered as the primary mover of cardiovascular responses. Generally, differences in translocated volume between subjects for the same box pressure are an intrinsic limitation of LBNP. Surprisingly few studies have addressed study paradigms enabling individualization LBNP-induced central blood volume shifts. We propose leg (blood) volume (Truijen et al., 2012a) as a controller of input to a sub-atmospheric pressure feedback loop. The finding that resting cardiovascular variables (including SV, HR, and MAP) were similar between consecutive trials, suggest that 5 min of rest is sufficient to reverse LBNP-induced fluid shifts.

Intra-subject reproducibility has been assessed in the present study by two different methods of reliability testing. We found that the results of both tests were contradictory for arterial blood pressure responses: poor vs. good reproducibility. Using ICCs can lead to inaccurately low reliability measurements as it is highly sensitive for the spread of the data whereas typical error (e.g., CV) is not (Hopkins, 2000). Several researchers reported (highly) reproducible blood pressure responses to repeated LBNP trials (Lightfoot et al., 1991; Convertino, 2001; Kay and Rickards, 2015) suggesting that CV is a more robust and accurate measure of reliability.

A sudden blood pressure drop observed in two subjects (one from group A and one from group B) during the last LBNP trial of that session emphasizes that we do not know whether blood pressure control would have been maintained beyond the applied timeframe of simulated central hypovolemia. The finding that a decline in blood pressure is more likely to occur with accumulating exposure to LBNP conforms to previous research (Lightfoot and Tsintgiras, 1995; Hinds and Stachenfeld, 2010).

Sympathetic stimulation is considered to enhance specifically the inotropic condition and lusitropic properties of the healthy heart (Thomas, 2011). We acknowledge that in the physiological laboratory as well as in a clinical environment changes in intrinsic cardiac muscle properties during sympathetic activation usually go by unnoticed in part or in whole.

We did not evaluate MSNA respectively the sympathetic BRS response, which might provide further insight into the dichotomy.

This study does not answer the question whether or not the distinct cardiovascular response patterns would have remained consistent over months or years. Convertino reported that re-testing after 1 year delivered comparable cardiovascular responses to LBNP (Convertino, 2001). With aging the interaction between neural and hemodynamic factors changes with different sex effects which is expected to modify the cardiovascular response to LBNP on the long run (Hart et al., 2012).

### Summary

In summary, the present study demonstrated distinct and reproducible cardiovascular response patterns to sympathetic stimulation by central hypovolemia in young healthy adults. Differences in resting HR and BRS between subjects suggest individually programmed reflex strategies of autonomic blood pressure control which may contribute to the variance observed in cardiovascular reflex responses to central hypovolemia. The mechanisms responsible for this phenomenon and the extent to which they operate in other groups of subjects deserve attention.

## Author contributions

A-SGT Bronzwaer and J Verbree contributed to the experimental design, data acquisition, data analysis, data interpretation, and writing the manuscript. WJ Stok contributed to data analysis and manuscript revision. MA van Buchem and MJAP Daemen contributed to data interpretation and manuscript revision. MJP van Osch contributed to experimental design, data interpretation, and writing the manuscript. JJ van Lieshout supervised the study, contributing to the experimental design, data analysis, data interpretation, and writing the manuscript. All authors approved the final version of the manuscript.

## Funding

The authors thank the Rembrandt Institute of Cardiovascular Science for support.

### Conflict of interest statement

The authors declare that the research was conducted in the absence of any commercial or financial relationships that could be construed as a potential conflict of interest.
